# Experimental studies suggest differences in the distribution of thorax elasticity between insects with synchronous and asynchronous musculature

**DOI:** 10.1098/rsif.2023.0029

**Published:** 2023-04-05

**Authors:** Cailin Casey, Chelsea Heveran, Mark Jankauski

**Affiliations:** Mechanical and Industrial Engineering, Montana State University, Bozeman, MT 59717, USA

**Keywords:** insect flight, indirect actuation, indirect flight muscles, thorax elasticity

## Abstract

Insects have developed diverse flight actuation mechanisms, including synchronous and asynchronous musculature. Indirect actuation, used by insects with both synchronous and asynchronous musculature, transforms thorax exoskeletal deformation into wing rotation. Though thorax deformation is often attributed exclusively to muscle tension, the inertial and aerodynamic forces generated by the flapping wings may also contribute. In this study, a tethered flight experiment was used to simultaneously measure thorax deformation and the inertial/aerodynamic forces acting on the thorax generated by the flapping wing. Compared to insects with synchronous musculature, insects with asynchronous muscle deformed their thorax 60% less relative to their thorax diameter and their wings generated 2.8 times greater forces relative to their body weight. In a second experiment, dorsalventral thorax stiffness was measured across species. Accounting for weight and size, the asynchronous thorax was on average 3.8 times stiffer than the synchronous thorax in the dorsalventral direction. Differences in thorax stiffness and forces acting at the wing hinge led us to hypothesize about differing roles of series and parallel elasticity in the thoraxes of insects with synchronous and asynchronous musculature. Specifically, wing hinge elasticity may contribute more to wing motion in insects with asynchronous musculature than in those with synchronous musculature.

## 1. Introduction

Flying insects are a diverse group with astounding ranges in body mass (approximately micrograms to grams) [1,2], wingbeat frequency (15–1000 Hz) [3,4] and flying abilities (e.g. hovering, aerial manoeuvres and sustained flight) [5]. Most insects realize flight through ‘indirect actuation’, whereby the flight muscles pull directly on the exoskeletal shell surrounding the thorax rather than at the wing base. The thorax deformation is then transformed into flapping via a complex linkage called the wing hinge [6–8]. Indirect actuation is hypothesized to improve the energetic economy of flight by allowing elastic energy to be stored in the thorax between wing flaps [9]. Thorax elasticity is therefore of fundamental importance to efficient flight.

Beyond indirect actuation, some insects possess specialized flight muscles believed to enhance flight efficiency. Many insects have ‘asynchronous’ flight muscles, where one neurological signal generates several wingbeats [10]. This differs from the one-to-one ratio between muscle signalling and wingbeat observed in insects with ‘synchronous’ flight muscles [10]. Asynchronous muscle function is enabled by stretch activation, where the tension caused by elongating one set of flight muscles triggers the antagonistic set of muscles to activate, thereby allowing mechanical muscle activation between neurological signals [11]. For synchronous muscle to activate, calcium must be cycled across the sarcoplasmic reticulum, which requires both time and energy [12]. Asynchronous muscles’ ability to activate without an action potential removes the need for extensive sarcoplasmic reticulum and calcium pumping. This allows more space for muscle fibres and enables higher wingbeat frequencies [12].

The evolution of asynchronous muscle may be accompanied by other physiological adaptations as well. For example, wing density per unit area is generally higher in insects with asynchronous musculature compared to those with synchronous musculature [4]. As a result, the inertial forces generated by the flapping wings are believed to be higher in insects with asynchronous musculature [13]. Aerodynamic forces generated by the flapping wings may also vary between asynchronous and synchronous groups, but they are generally of the same order as the weight of the insect to produce sufficient lift for hover [14]. Unlike the flight muscle forces, which are applied at the dorsalventral and anterior/posterior thoracic walls, the aerodynamic and inertial forces generated by the flapping wing act at the fulcrum formed by the thoracic walls near the wing base.

**Figure 1.**
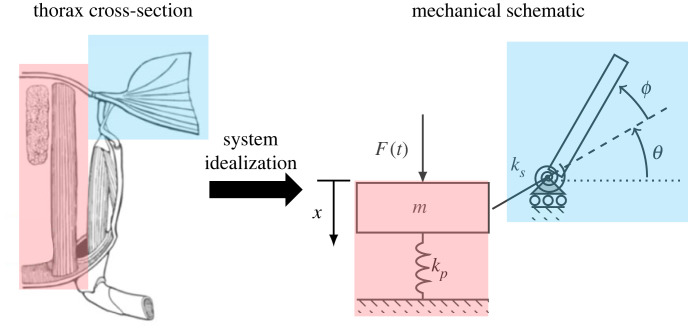
The insect thorax (left) can be idealized as a two-degree-of-freedom mechanical model (right). Within the mechanical model, the elasticity is lumped into two components: a parallel elastic element of stiffness $k_{p}$ that represents the flight muscles and the thorax exoskeleton (highlighted in red), and a series elastic element of torsional stiffness $k_{s}$ that represents the rotational elasticity of the wing hinge and the thorax exoskeleton immediately surrounding the wing base (highlighted in blue). The parallel element deforms a distance x  directly under muscle force F , which causes the wing to rigidly rotate an amount *θ*. The wing may rotate an additional amount *ϕ* about the wing hinge if the series element deforms under the inertial and aerodynamic loading of the wing. Left image adapted from [13,16].

Though it is often thought that muscle action is the dominant source of thorax deformation, wing aerodynamic and inertial forces may deform the thoracic exoskeleton locally at the fulcrum. Consequently, recent studies have imagined the thorax as a system with elasticity distributed in two primary regions: a parallel elastic element, representative of the combined elasticity of the primary flight muscles and/or the compliant thorax walls they act on, and a series elastic element, representative of the (usually rotational) elasticity of the wing hinge and the thoracic exoskeleton immediately surrounding the wing base (figure 1) [13]. Mathematical modelling has demonstrated the importance of both elastic elements to flight energetics [15], though there are few experimental studies that address elasticity distributions in real insects.

Despite advances in our understanding of insect thorax mechanics and muscle physiology, there remain the questions: how is elasticity distributed throughout an insect thorax, and does this distribution differ between insects with synchronous and asynchronous flight musculature? The goal of the current study is to provide evidence for elasticity distributions in these two insect groups. Within this work, thorax elasticity is assumed to be lumped into series and parallel elements to remain consistent with recent modelling efforts [13,15]. The stiffness of the parallel elastic element can be measured directly via force–displacement testing on sacrificed insects. Conversely, the series element is more challenging to measure directly, as many insects disengage their wings post-sacrifice. This renders the moment–angular displacement tests that are necessary to assess series element stiffness inaccessible.

The two studies presented here estimate elasticity distributions in the thoraxes of insects with synchronous and asynchronous flight musculature. In the first study, thorax deformations and aerodynamic/inertial forces generated by the flapping wings are simultaneously measured in tethered flying insects. While this study does not directly measure stiffness of the series elastic element, it provides a quantitative measure of the forces that deform this element. Assuming that the wing hinge region and surrounding exoskeletal cuticle are similarly stiff across insects, the series elastic element will deform more when wing-generated forces are larger. While difficult to assess this assumption directly, thorax cuticle modulus is similar between hawkmoths and honeybees, which have synchronous and asynchronous flight musculature, respectively [17]. Thorax cuticle normalized by thorax diameter is similar between these species as well (unpublished data). This provides evidence that the relative series element stiffness is similar across insects, though additional studies that account for wing hinge soft tissue material properties and geometry are required to address this quantitatively. Within this work, the total wing-generated forces will be used as a proxy for the relative influence of the series element. A second study is used to directly measure the stiffness of the parallel elastic element in multiple insect species. Insect thoraxes are quasi-statically compressed using the deformation amplitudes measured during the first study and the stiffness of the parallel elastic element is estimated via the slope of the resulting force–displacement curve. Together, these studies provide evidence that the relative influence of parallel and series elasticity in flapping wing insects differs between those with synchronous and asynchronous flight muscles.

## 2. Methods

### 2.1. Specimen collection and care

Experiments were conducted on six insect species with synchronous and asynchronous musculature that spanned two orders of magnitude in mass (approx. 10^−2^ to 10^0^ grams). Species with asynchronous musculature included *Bombus centralis* (bumblebee), *Xylocopa californica* (carpenter bee) and *Musca domestica* (housefly). Species with synchronous musculature included *Manduca Sexta* (hawkmoth), *Helicoverpa zea* (corn earworm moth) and *Agrotis ipsilon* (black cutworm moth).

*B. centralis* specimens were collected locally in Bozeman, MT. Specimens were tested within 3 h of capture. *X. californica* specimens were collected in Tuscon, AZ and shipped overnight to Montana State University in Bozeman, MT with a damp sponge. They were refrigerated at 4°C and were tested within 3 days of arrival. *M. domestica*, *A. ipsilon* and *H. zea* pupae were shipped overnight from Benzon Research (Carlisle, PA, USA), and *M. Sexta* pupae were shipped overnight from University of Washington (Seattle, WA, USA). Moth and housefly pupae were housed in a rearing box consisting of a plastic tub filled with damp paper towels or peat moss with gutter mesh on the sides for climbing to facilitate wing unfurling. The box was kept at 24 : 0 light : dark cycle to avoid diapause in Lepidoptera. Specimens were tested within 4 days of emergence.

### 2.2. Tethered flight testing

Since series elasticity cannot easily be measured in flying insects, the wing-generated forces are used as a proxy to estimate the relative influence of series elasticity on wing actuation. Wing-generated forces include aerodynamic forces generated by fluid pressure acting on the wing surface and inertial forces associated with the rapid acceleration and deceleration of the wing mass. A tethered flight study is devised to simultaneously measure these wing-generated forces and the deformation of the thorax in the dorsalventral direction. Though tethering is known to influence the behaviour of flying insects, for example lowering wingbeat frequency [17–19], it is necessary to isolate thorax deformation from body motion (e.g. periodic body translations during hovering) and to measure wing-generated forces. Wing-generated forces act at the exoskeletal fulcrum near the wing base but are propagated to the dorsal thorax where they can be measured using an attached force sensor. Though the force sensor may also record DVM muscle forces, these forces are expected to be small; the DVM muscles exert an equal and opposite force on the upper and lower thorax exoskeletal walls, so the net force at the tether resulting from DVM action should be nearly zero. Because the main forces being measured are back propagated from the wing hinge, the aggregate forces measured at the tether will be referred to as ‘wing-generated forces’ hereafter.

**Figure 2.**
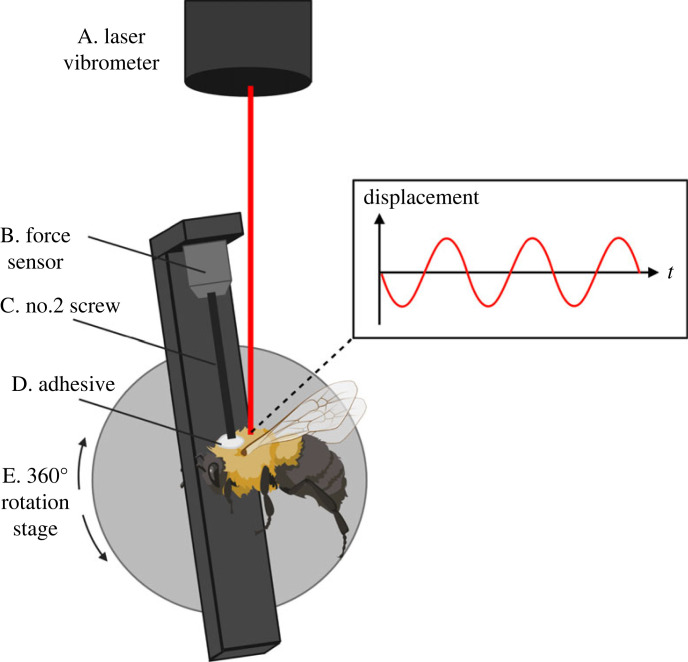
*In vivo* tethered flight experimental set-up. A laser vibrometer measures the velocity of the dorsal thorax (numerically integrated to find displacement), and a piezoelectric force sensor measures wing-generated forces at the tether location. The rotation stage is used to align the thorax to improve reflectance to the vibrometer.

Wing-generated forces and velocity at the dorsal thorax are measured in tethered flying insects using a piezoelectric force sensor and laser vibrometer, respectively (figure 2). A laser vibrometer (VibroGo VGO-200, Polytec, Baden-Württemberg, Germany) was attached to a metal plate between two support beams so that the laser was directed downward to measure velocity at the dorsal thorax. Thorax velocity was subsequently integrated numerically to determine thorax displacement. Below, a rotation stage was mounted to a support on a translation stage to facilitate suspension and proper alignment of the insects. A custom three-dimensional-printed chopped carbon fibre bracket (MethodX, MakerBot, Brooklyn, NY, USA) was attached to the rotation stage on one side and a force sensor (209C11, PCB Piezotronics, Depew, NY, USA, sensitivity 494 604 mV kN^−1^) was attached to the other. A piezoelectric force sensor was specifically chosen for this experiment because of its high natural frequency (compared to foil-based load cells), large measurement range (0.5–30 000 Hz) and excellent force resolution. This allowed for a single force transducer to be used across all insect species studied. However, piezoelectric force sensors are AC coupled and cannot measure static forces. Consequently, the insect weight and constant component of aerodynamic lift are excluded from the force measurement, though the oscillatory component of the aerodynamic force remains. The effective resonant system of the tethering system, including the force sensor, is about 510 Hz, over three times greater than the highest wingbeat frequency of any insect considered in this study.

A LabVIEW program was written to collect data from the vibrometer and force sensor. Data were sampled at 5000 measurements per second. A compact DAQ was used to synchronize measurements (cDAQ-9178, National Instruments (NI), Austin, TX, USA). DAQ modules NI 9215 and NI 9230 were used for the laser vibrometer and the force sensor, respectively.

Specimens were cold anaesthetized in a freezer at −18°C until quiescent (about 30 s to 8 min depending on the species) and then massed (XS603S, Mettler Toledo, Columbus, OH, USA). For Lepidoptera, thorax scales were removed with a scalpel and damp paper towels. For all species except *M. sexta*, hot glue was used to adhere the dorsal surface of the thorax to the tether. *M. sexta* produced forces large enough to liberate themselves from the tether when hot glue was used as adhesive. Instead, cyanoacrylate was used to attach a 1/8′ disc magnet to the anterior tergum. The tergum was then magnetically attached to the force sensor.

Because the scutellum (the thorax posterior) is the primary location of thorax deformation in Diptera and Hymenoptera [20,21], the force sensor was attached to the thorax anterior for insects in these orders. Lepidoptera were secured to the force sensor at the thorax posterior, where thorax deformation is lower compared to the anterior [22]. This reduced the amount of body motion recorded and ensured that vibration measurements were taken where the thorax deformation was considerable. The laser was focused on the centre of the unconstrained portion of the thorax. Due to slight differences in gluing, exact laser placement varies slightly, though laser placement is not believed to contribute significantly to the variation in displacement measurements. Previously reported spatial measurements of the dorsal thorax displacement in hawkmoths showed that thorax displacement is largest at the wing base (0.4 mm) and smallest at the centre of the thorax (0.15–0.2 mm) [22]. In the present study, displacement at the centre thorax for the hawkmoth *M. sexta* was similar in magnitude to reported measurements taken at the same location (see Results). Spatial distribution of thorax deformation is not available for other species studied here, but the relatively narrow ranges in displacement (compared to the relatively large range in displacements across the thorax of an individual) suggest that laser placement is likely not a significant factor for any species.

### 2.3. Tethered flight analysis

**Figure 3.**
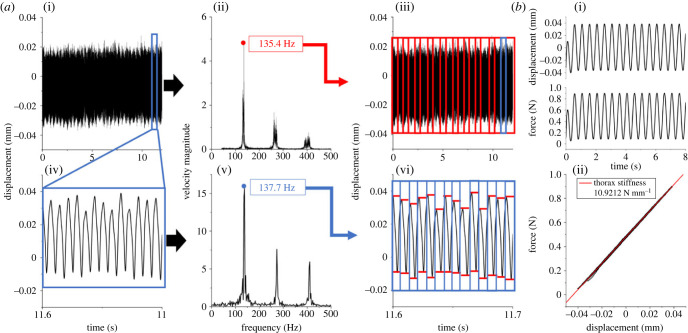
Example of data analysis from *B. centralis* for (*a*) *in vivo* flight data and (*b*) dorsalventral thorax stiffness. (*a*) Data are shown for both the entire flight period (i*–*iii) and a single segment within the flight period (iv–vi). First, time-series data for the entire flight duration (i) were transformed using FFT to identify the average wingbeat frequency (ii). Wingbeat frequency was used to divide the flight period into smaller segments for more refined analysis while maintaining a frequency resolution of 1.5% wingbeat frequency (iii). Since wingbeat frequency was variable over the flight period, this process was repeated for each segment (iv–vi). Wingbeat frequency (v) was used to divide each segment into individual wingbeats where the peak–peak values for displacement and total forces were measured using a peak finding algorithm. Peak-to-peak distances were averaged for each segment, and again for the entire flight segment and then converted to amplitudes. (*b*) The thorax was compressed at 2 Hz for at least 10 cycles (i). Linear regression for force versus displacement was used to determine the average dorsalventral stiffness (ii).

Collected data were analysed to determine wing-generated force and displacement amplitudes as well as wingbeat frequency (assumed to coincide with the thorax oscillation frequency). Because these parameters may vary over time [9,23,24], the flight period was divided into segments. The fast Fourier transform (FFT) of thorax velocity was taken for each segment. Since FFT frequency resolution and segment length are inversely related [25], the segment length was calculated to maintain a resolution of 1.5% of the wingbeat frequency for each flight trial (figure 3*a*(i–iii)). Band pass filters were applied to the data, filtering data from 1/3 to 3 times the minimum and maximum wingbeat frequency measured during a trial (e.g. for a wingbeat frequency ranging from 30 to 35 Hz, the band pass filter was applied between 10 and 105 Hz). For each segment, the number of wingbeats was estimated based on wingbeat frequency and segment duration (figure 3*a*(iv–vi)). For each wingbeat, thorax deformation and wing-generated force amplitudes were calculated. Finally, deformation and wing-generated force data were averaged.

To compare data between species of disparate size, wing-generated force and thorax deformation were non-dimensionalized to insect body weight and thorax diameter, respectively. Thorax diameter was measured with digital calipers in the sagittal plane. Sagittal plane thorax diameter was used in lieu of more traditional length-scale measurements (e.g. intertegular distance) because it more closely relates with the resting length of the DVM muscle group. Trials where relative wing force amplitude was greater than 10 times body weight for insects with synchronous musculature, 20 times body weight for insects with asynchronous musculature and/or relative deformation amplitude greater than 5% thorax diameter were removed from analysis. Since insects with asynchronous muscle generally experienced larger wing-generated forces, the cutoff value was higher for those species. Values of this magnitude were associated with poor adherence between the thorax and the force sensor and thus increased body movement, thereby affecting wing-generated forces and displacement values.

### 2.4. Quasi-static thorax force–displacement

**Figure 4.**
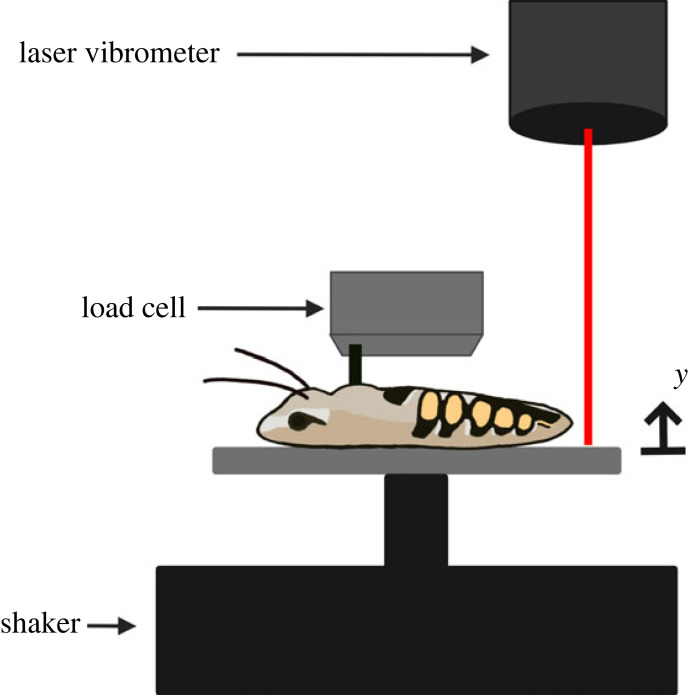
Thorax compression experimental set-up. The thorax is compressed sinusoidally at 2 Hz with *in vivo* amplitudes, and the resulting compressive forces are measured at the dorsal thorax. Dorsalventral thorax stiffness is estimated from the resulting force–displacement curves. ‘*y*’ denotes thorax deformation.

To estimate the stiffness of the parallel elastic element (figure 1), dorsalventral thorax stiffness was measured via quasi-static force–displacement testing of all species considered during tethered flight studies (figure 4). Specimens were sacrificed using ethyl acetate in a kill jar and all tests were completed within 20 min of sacrifice. Prior to testing, the wings and legs were removed from all cadavers. Scales were removed from the Lepidoptera thorax to better visualize the tergal plate. Cadavers were glued to a surface by the ventral thorax. Glue was left to dry for 2–5 min.

A vibration shaker (2007E, The Modal Shop, Cincinnati, OH, USA) was used to impose a 2 Hz sinusoidal oscillation to the thorax. Compression at 2 Hz was fast enough to avoid relaxation behaviour but slow enough to avoid dynamic effects. Foil load cells (GSO series models 100 and 10, depending on species, Transducer Techniques, Temecula, CA, USA) were used to measure forces at the dorsal portion of the thorax, near where forces were measured during tethered flight studies. Thorax deformation was indirectly measured via a laser vibrometer recording the stage displacement where the insect was fixed.

The dorsal thorax surface was located by lowering the foil cell until the force recording increased. The thorax was then pre-compressed to about 50% of the deformation amplitude measured *in vivo* such that the sensor did not lose contact with the thorax at peak displacement. For each species, thorax compression amplitude was based on the largest thorax displacement (mm) measured during the *in vivo* flight trials. Sensor position was finely adjusted to ensure that the sensor maintained contact with the thorax during testing as the thorax was cycled from maximum compression to its resting, undeformed position. The thorax was compressed approximately 10 cycles about its pre-compressed state (figure 3*b*(i)). Dorsalventral thorax stiffness was estimated by calculating the slope of the linear regression of the steady-state force versus displacement data (figure 3*b*(ii)). Quasi-static thorax stiffness measurements were non-dimensionalized in the same way that thorax displacement and force were non-dimensionalized in tethered flight so that the results of the two studies could be compared.

### 2.5. Statistics

Because flight duration and wingbeat frequency varied greatly between individuals and species, preliminary general linear models were used to test the impact of the number of wingbeats measured and wingbeat frequency, thorax displacement, and wing-generated forces. Next, general linear models were used to assess the impact of species and muscle type on *in vivo* flight measurements and quasi-static thorax stiffness. For *in vivo* flight data, relative force and displacement were averaged for each specimen to avoid overpowering the statistical results. For specimens where multiple trials were collected, the flight data were again averaged to report one value for displacement, force and wingbeat frequency per specimen. For stiffness data, three trials were averaged per specimen. In all models, muscle type and species were assigned as fixed factors with species nested in muscle type. Data were natural log transformed when needed to satisfy the assumptions of homoscedasticity and residual normality. *Post hoc* testing for species, when significant, was performed using a Tukey test. Tests were performed using Minitab v. 19 2020 2.0. The threshold for significance was set *a priori* at *p* < 0.05.

## 3. Results

### 3.1. Tethered flight

**Figure 5.**
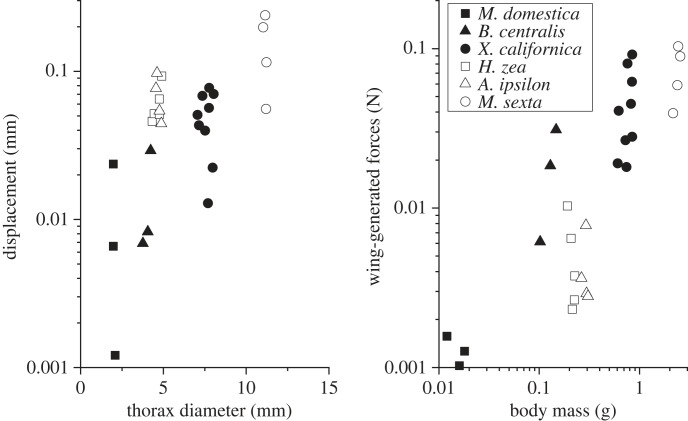
Scatter plots for average displacement (mm) versus thorax diameter (mm) and wing-generated force (N) versus body mass (g) by specimen. Shapes indicate species and black/white indicates asynchronous and synchronous muscle, respectively.

**Figure 6.**
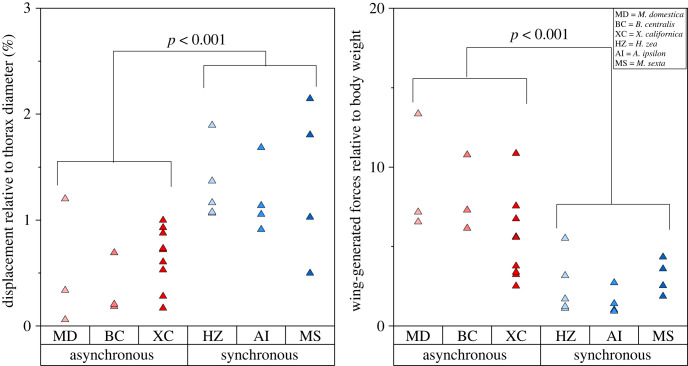
Individual value plots for specimen. Thorax displacement is relative to thorax diameter and wing force is relative to body weight. Values of *p* refer to the effect of muscle type from associated two-factor ANOVA models.

For each of the six species, three–nine specimens were tested, with 3–15 total trials recorded for each species. Flight duration and wingbeat frequency varied between species and individual specimens, leading to trials with a range of 75 wingbeats to 7600 wingbeats captured. Three independent general linear models provided compelling evidence that the average number of wingbeats analysed did not affect the response variables: wingbeat frequency, thorax displacement and wing-generated forces with *p* = 0.271, 0.771 and 0.692, respectively. Flight data were again averaged to report one value for displacement, force and wingbeat frequency for each specimen (table 1). For all insects, absolute thorax deformation and wing forces were positively correlated with thorax diameter and body mass, respectively (figure 5). To compare across species, force relative to body weight and displacement relative to thorax diameter were considered. For all insects, mean thorax displacement was in the range of 0.4–1.4% of thorax diameter (table 1). Insects with synchronous musculature generally had relative thorax deformation ranging from 1.2 to 1.4% while insects with asynchronous musculature had relative deformations ranging from 0.4 to 0.7%. For relative displacement, muscle type (*p* < 0.001) but not species (nested in muscle type, *p* = 0.782) significantly affected mean relative thorax displacement. On average, the species with asynchronous musculature had 60% smaller relative thorax displacement. For relative force, muscle type (*p* < 0.001) but not species (nested in muscle type, *p* = 0.091) significantly affected mean force (figure 6). Insects with asynchronous musculature experienced wing-generated forces between 5.5 and 9.0 times their body weight, while insects with synchronous musculature produced forces between 2.5 and 3.1 times greater than their body weight. On average, the species with asynchronous musculature had 2.8 times greater wing-generated forces.

**Table 1.** Descriptive statistics for flight data. All values are mean ± standard deviation.

species	*N* specimens	*n* trials (per species)	average number of wingbeats per trial	body mass (g)	thorax diameter (mm)	frequency (Hz)	displacement (mm)	wing-generated force (N)	displacement relative to thorax diameter (%)	total force relative to body weight
*M. domestica*	3	3	2813 ± 4169	0.015 ± 0.069	2.01 ± 0.07	140.8 ± 11.0	0.010 ± 0.002	0.0013 ± 0.0003	0.53 ± 0.60	9.02 ± 3.76
*B. centralis*	3	5	4020 ± 2474	0.126 ± 0.023	4.02 ± 0.23	160.0 ± 3.9	0.015 ± 0.012	0.0103 ± 0.0048	0.36 ± 0.29	8.07 ± 2.40
*X. californica*	9	15	555 ± 491	0.755 ± 0.094	7.59 ± 0.35	104.5 ± 12.1	0.049 ± 0.021	0.0373 ± 0.0227	0.65 ± 0.28	5.47 ± 2.64
*H. zea*	5	10	214 ± 180	0.214 ± 0.014	4.63 ± 0.24	37.3 ± 5.5	0.061 ± 0.019	0.0051 ± 0.0033	1.31 ± 0.35	2.54 ± 2.65
*A. ipsilon*	4	7	702 ± 423	0.290 ± 0.018	4.70 ± 0.15	29.3 ± 5.6	0.056 ± 0.014	0.0043 ± 0.0024	1.20 ± 0.34	2.54 ± 1.86
*M. sexta*	4	7	229 ± 159	2.379 ± 0.164	11.15 ± 0.09	16.7 ± 0.8	0.152 ± 0.082	0.0728 ± 0.0290	1.36 ± 0.75	3.08 ± 1.10

Tethering may have reduced wingbeat frequencies across all species when compared to known wingbeat frequencies for the same species or the similar species with available wingbeat frequency data. *B. terrestris* has a free flight wingbeat frequency of 160–180 Hz [26]. Therefore, the average wingbeat frequency of 160 Hz for *B. centralis* measured in this study was on the low end of the expected range. *M. domestica*, *X. californica*, *A. ipsilon* and *H. zea* had wingbeat frequencies lower than their expected ranges (141 versus 144–170 Hz) [27,28], (105 versus 115–130 Hz for *X. varipuncta*) [29], (29 versus 37–42 Hz) [30] and (37 versus 44–52 Hz for *H. armigera*) [30], respectively. The greatest discrepancy was for *M. sexta* which had a mean wingbeat frequency of 17 Hz in this study while free-flying individuals generally have a wingbeat frequency of 25 Hz [31].

### 3.2. Quasi-static thorax stiffness

Assuming the thorax can be idealized as the distributed elastic model discussed previously, the stiffness of the parallel elastic element is well approximated by the stiffness of the thorax in the dorsalventral direction. The dorsalventral thorax stiffness was directly measured on the same species measured during *in vivo* flight. Mean compression amplitudes (mm) were based on the largest mean displacement measured for individual specimen during tethered flight for each species: 0.035 for *M. domestica*, 0.036 for *B. impatiens*, 0.097 for *X. californica*, 0.099 for *H. zea*, 0.134 for *A. ipsilon*, 0.246 for *M. sexta*. *X. californica*, *M. Sexta, A. ipsilon* and *H. zea* were measured with GSO-100; while *B. impatiens* and *M. domestica* were measured with GSO-10. GSO suffix denotes the maximum load cell capacity in grams. Although these compression values may exceed the maximum compression for an individual insect, all thoraxes behaved linearly during force–displacement testing, and consequently the compression amplitude will not affect stiffness. Across all force–displacement tests, the linear fit used to identify stiffness had a minimum *R*^2^ value of 0.95, which substantiated the linear assumption.

**Figure 7.**
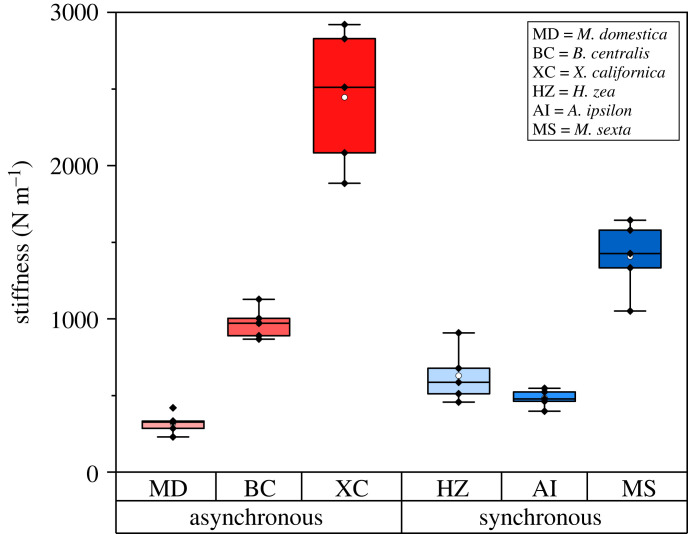
Absolute thorax stiffness (N m^−1^) for all species considered (*N* = 5 for each species). Mean indicated by a white circle.

**Figure 8.**
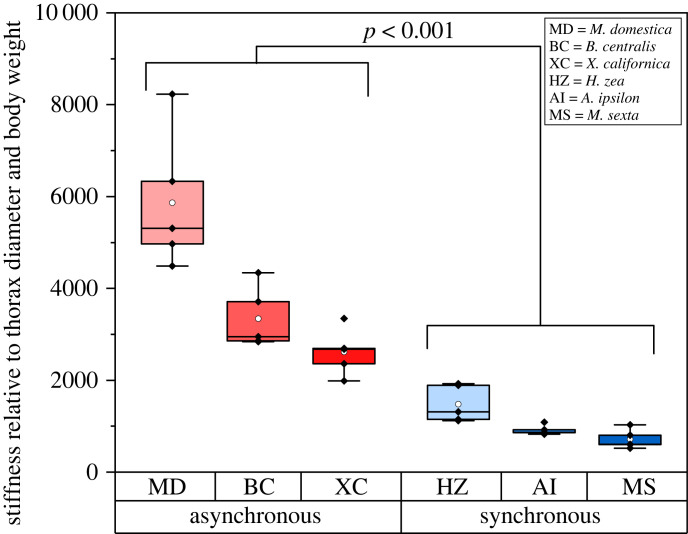
Thorax stiffness relative to body weight and thorax diameter for each species (*N* = 5 for each species). Mean indicated by a white circle.

Within each muscle type, thorax stiffness depended on size, with larger insects having stiffer thoraxes (figure 7). To compare across species, stiffness (N m^–1^) was non-dimensionalized by body weight and thorax diameter (figure 8). Thorax stiffness was non-dimensionalized in the same way as that for *in vivo* flight measurements to make results between the two experiments comparable. Thoraxes for insects with asynchronous musculature had higher relative stiffness than insects with synchronous musculature (*p* < 0.001). On average their thoraxes were 3.8 times stiffer than for insects with synchronous muscle. Relative thorax stiffness did depend on species (*p* < 0.001). For insects with asynchronous musculature, relative thorax stiffness for *M. domestica* was significantly higher than for *B. impatiens* and *X. californica*. For insects with synchronous muscle, relative thorax stiffness for *H. zea* was significantly higher than for *M. sexta* and *A. ipsilon*.

## 4. Discussion

Most flying insects achieve flight via indirect actuation, where the thorax is deformed via flight muscles and thorax deformations are amplified into wing rotation by an intermediate linkage. Thorax elasticity and deformations are believed to contribute to the energy efficiency of flight and have garnered significant interest in recent years. Recent modelling efforts have treated the thorax as a flexible system with elasticity distributed across two elements (figure 1). These include a parallel element (representative of the elasticity of the flight muscles and bulk exoskeletal cuticle) and a series element (representative of the compliance of the wing hinge). This paper focused on estimating the elastic contribution of the series and parallel elastic components proposed during recent thorax modelling efforts [13,15]. First, the aerodynamic and inertial forces that flapping wings produced during tethered flight were used as a proxy for the contribution of the series elastic element in wing actuation. Second, thorax dorsalventral stiffness was used as a direct measure of the parallel elastic element. Species with synchronous and asynchronous musculature were tested to compare the elastic distribution between insects with these two muscle types.

### 4.1. Relative thorax deformation and forces

The first experiment measured wing-generated forces and thorax deformation at the dorsal thorax. Relative to their size, insects with asynchronous musculature deformed their thorax less than insects with synchronous musculature (*p* < 0.001). On average, relative thorax deformations were about 60% smaller in the asynchronous group (table 1 and figure 6). This difference in relative thorax deformation is comparable to reported differences in flight muscle strains between *Bombus* species (asynchronous muscles; 1–3%) [32–34] and *M. sexta* (synchronous muscles; 5.5–8.6%) [35–37].

The relative thorax displacements measured here were generally smaller than reported flight muscle strains. Relative thorax deformations of about 0.4% and 1.7% in *B. centralis* and *M. sexta,* respectively, were about 60–85% lower than flight muscle strains reported for the same species. A discrepancy between muscle strain and relative exoskeletal deformation may occur if the thorax deforms more significantly at the muscle attachment sites than it does at the medial line where deformations were measured in the present study. Time-resolved profileometer studies show that the dorsal thorax deforms non-uniformly on the hawkmoth *Agrius convolvuli*, where deformation is smallest near the medial line and increases towards the wing base near where the DVM muscle group attaches [22]. Although the thorax deformation distribution is unknown in other insect species, it is likely that most thoraxes deform non-uniformly. There remains an opportunity to map the spatio-temporal deformation field of the dorsal thorax in other insect species to expand upon the single-point deformation measurements considered here.

Wing-generated forces were about three times higher in insects with asynchronous flight musculature compared to insects with synchronous flight musculature when accounting for insect weight (table 1 and figure 6). Relative aerodynamic force amplitudes are expected to be approximately equal to body weight for all insects [38], so differences in wing-generated forces between the asynchronous and synchronous groups likely stem from differences in inertial forces. Inertial forces are at a maximum in the stroke plane, which varies by species and flight behaviour [31,39,40]. The force sensor measures forces only in the vertical direction so inertial forces may be underestimated. Based on scaling laws, insects like those used in this study with asynchronous musculature are expected to have inertial forces that are approximately 5.5 times larger than their aerodynamic forces, while insects with synchronous musculature are expected to have inertial forces that are approximately 2.2 times larger than their aerodynamic forces [4]. If aerodynamic forces are assumed to have an amplitude equivalent to the insect weight in the current study, this suggests the inertial forces have amplitudes about 1.5–2 times larger than the insect weight and 4–8 times larger than the insect weight in insects with synchronous and asynchronous muscle, respectively (table 1). This provides evidence to support the inertial-to-aerodynamic force ratios reported in earlier studies based on scaling laws [4] and suggests that the wing hinge region experiences larger relative forces in insects with asynchronous muscle.

### 4.2. Dorsalventral thorax stiffness

In a second experiment, the dorsalventral thorax stiffness was measured to directly characterize the parallel elastic element. Thorax stiffness was comparable to values reported in the literature. Stiffness measured for *M. sexta* was between previously reported values of 620 N m^–1^ in the dorsalventral direction [41] and 3750 N m^–1^ in the dorsal-longitudinal direction [9]. Note that [41] did not consider the influence of stress relaxation, so 620 N m^–1^ is likely an underestimate of dorsalventral stiffness. To our knowledge, thorax stiffness has not been directly measured in any other species. Dorsalventral thorax stiffness was higher in insects with asynchronous musculature when accounting for size and weight (*p* < 0.001). On average, the relative stiffness of thoraxes with asynchronous muscle was 3.8 times stiffer than for thoraxes with synchronous muscle (figure 7).

### 4.3. The effects of distributed elasticity

Collective findings from the two experiments provide insight into the central questions: how is elasticity distributed throughout an insect thorax and does this distribution differ between insects with synchronous and asynchronous flight musculature? It is hypothesized here that deformation of the series element contributes more to wing motion insects with asynchronous flight muscle compared to those with synchronous flight muscle. Conversely, deformation of the parallel element contributes more to wing motion in insects with synchronous flight muscle compared to those with asynchronous muscle.

Relative to their size, insects with asynchronous musculature have stiffer thoraxes compared to insects with synchronous musculature, which indicates a higher stiffness of the parallel elastic element (figure 8). Increased thorax stiffness may stem from the higher passive stiffness of the asynchronous flight muscle [10] or differences in geometric properties of the thoracic exoskeleton surrounding the flight muscle, since the material properties of the thorax cuticle appear to be similar across insect species [42]. Further, the wing-generated forces acting at the exoskeletal fulcrum are higher in insects with asynchronous muscle due to an increase in wing inertia (figure 6). Though the stiffness of the series elastic element is unknown, large forces acting at wing base suggest larger deformation of the series elastic element, and hence greater wing rotation. Because the wing is free to rotate beyond the amount governed by thorax deformation, the thorax must deform relatively little to generate large wing rotations. Indeed, thoraxes of insects with asynchronous muscle deform relatively less compared to those with synchronous muscle (figure 6).

What is the benefit of a flight configuration with high-parallel–low-series stiffness? Insects with asynchronous flight musculature are believed to flap at or near the resonance frequency of their thorax–wing system [10]. When flapping in proximity to resonance, the muscle forces required to deform the parallel element are relatively low, even if the parallel element is stiff. The reduced forces and low muscle strains may reduce the energetic expenditures required during flight. At the same time, the insect must remain near resonance to realize these energetic benefits. Deviation in frequency or phase is believed to incur an energetic penalty [43], so it is more beneficial for the thorax to maintain a constant oscillatory rhythm. This is likely why steering in insects with asynchronous muscle is achieved via small synchronous muscles embedded within the wing hinge rather than by the flight muscles themselves [44], which would also benefit from a more compliant wing hinge area.

Contrasting insects with asynchronous muscle, those with synchronous muscle have less stiff thoraxes and, consequently, lower stiffness of the parallel elastic element (figure 8). Again, this may result from lower passive stiffness of the synchronous flight muscle [10]. The wing-generated forces tend to be lower (figure 6) because of reduced wing inertia [4]. Under this configuration, the deformation of the series elastic element may be smaller, and thus the rotation of the wing may be statically related to thorax displacement. This would require the thorax in insects with synchronous muscle to deform more than those with asynchronous muscles, which was observed in this study (figure 6). A low-parallel–high-series stiffness flight configuration has benefits as well. Unlike insects with asynchronous flight muscle, those with synchronous flight muscle are hypothesized to flap in a post-resonant regime [43]. As a result, deviation from their normal wingbeat frequency does not suffer as appreciable an energetic cost. The implication of this is that the flight muscles themselves may be used to manoeuvre in insects with synchronous muscle. Recent studies have shown that indirect flight muscles in *A. convolvuli* can indeed induce pitching corrections when the insect is subject to simulated optical disturbances [22]. In this case, it is desirable that the series element is sufficiently stiff such that the wing kinematics adapt instantaneously to the change in muscle kinematics. It is also desirable that the parallel element is flexible such that lower muscle forces are required to deform the thorax, since the flight muscles may be operating at a frequency off resonance.

In reality, the degree to which series and parallel stiffness contribute to thorax stiffness for different insects is unknown, but it is likely to be a continuum ranging from a very stiff series elastic element and a very compliant parallel elastic element to a very stiff parallel elastic element and a very compliant series elastic element. Although the contributions from the series and parallel elastic elements are likely not a binary divided by muscle type [15], this work supports an argument for insects with synchronous musculature generally having a stiffer series elastic element, and the parallel elastic element playing a larger role in wing actuation, and the opposite being true for insects with asynchronous musculature.

## Data Availability

Summary data for *in vivo* flight experiments and thorax compression experiments have been uploaded as part of the electronic supplementary material [45].

## References

[1] Ellington CP. 1999 The novel aerodynamics of insect flight: applications to micro-air vehicles. J. Exp. Biol. **202**, 3439-3448. (10.1242/jeb.202.23.3439) 10562527

[2] Setsuda K, Tsuchida K, Watanabe H, Kakei Y, Yamada Y. 1999 Size dependent predatory pressure in the Japanese horned beetle, *Allomyrina dichotoma* L. (Coleoptera; Scarabaeidae). J. Ethol. **17**, 73-77. (10.1007/BF02769300)

[3] Sotavalta O. 1953 Recordings of high wing-stroke and thoracic vibration frequency in some midges. Biol. Bull. **104**, 439-444. (10.2307/1538496)

[4] San Ha N, Truong QT, Goo NS, Park HC. 2013 Relationship between wingbeat frequency and resonant frequency of the wing in insects. Bioinspir. Biomim. **8**, 046008. (10.1088/1748-3182/8/4/046008) 24166827

[5] Hedrick TL, Combes SA, Miller LA. 2015 Recent developments in the study of insect flight. Can. J. Zool. **93**, 925-943. (10.1139/cjz-2013-0196)

[6] Pringle S. 1948 Zoology, pp. 226-232. Cambridge, UK: University of Cambridge.

[7] Nachtigall W, Wisser A, Eisinger D. 1998 Flight of the honey bee. VIII. Functional elements and mechanics of the ‘flight motor’ and the wing joint—one of the most complicated gear-mechanisms in the animal kingdom. J. Comp. Physiol. B **168**, 323-344. (10.1007/s003600050152)

[8] Rheuben MB, Kammer AE. 1987 Structure and innervation of the third axillary muscle of *Manduca* relative to its role in turning flight. J. Exp. Biol. **131**, 373-402. (10.1242/jeb.131.1.373) 3694116

[9] Gau J, Gravish N, Sponberg S. 2019 Indirect actuation reduces flight power requirements in *Manduca sexta* via elastic energy exchange. J. R. Soc. Interface **16**, 20190543. (10.1098/rsif.2019.0543) 31847756 PMC6936040

[10] Josephson RK, Malamud JG, Stokes DR. 2000 Asynchronous muscle: a primer. J. Exp. Biol. **203**, 2713-2722. (10.1242/jeb.203.18.2713) 10952872

[11] Pringle JWS. 1949 The excitation and contraction of the flight muscles of insects. J. Physiol. **108**, 226-232. (10.1113/jphysiol.1949.sp004326) 16991854 PMC1392366

[12] Syme DA, Josephson RK. 2002 How to build fast muscles: synchronous and asynchronous designs. Integr. Comp. Biol. **42**, 762-770. (10.1093/icb/42.4.762) 21708773

[13] Lynch J, Gau J, Sponberg S, Gravish N. 2021 Dimensional analysis of spring-wing systems reveals performance metrics for resonant flapping-wing flight. J. R. Soc. Interface **18**, 20200888. (10.1098/rsif.2020.0888) 33593213 PMC8086844

[14] Berman GJ, Wang ZJ. 2007 Energy-minimizing kinematics in hovering insect flight. J. Fluid Mech. **582**, 153-168. (10.1017/S0022112007006209)

[15] Pons A, Beatus T. 2022 Distinct forms of resonant optimality within insect indirect flight motors. J. R. Soc. Interface **19**, 20220080. (10.1098/rsif.2022.0080) 35582811 PMC9114943

[16] Snodgrass RE. 1935 Principles of insect morphology, pp. 510-549. New York, NY: McGraw Hill.

[17] Baker PS, Gewecke M, Cooter RJ. 1981 The natural flight of the migratory locust, *Locusta migratoria* L. J. Comp. Physiol. **141**, 233-237. (10.1007/BF01342669)

[18] Gewecke M. 1975 The influence of the air-current sense organs on the flight behaviour of *Locusta migratoria*. J. Comp. Physiol. **103**, 79-95. (10.1007/BF01380046)

[19] Fry SN, Sayaman R, Dickinson MH. 2005 The aerodynamics of hovering flight in *Drosophila*. J. Exp. Biol. **208**, 2303-2318. (10.1242/jeb.01612) 15939772

[20] Boettiger EG, Furshpan E. 1952 The mechanics of flight movements in Diptera. Biol. Bull. **102**, 200-211. (10.2307/1538368)

[21] Pfau HK. 2020 Functional morphology of the metathorax and hind wing of *Apis mellifera* (Hymenoptera: Apidae). Adv. Anim. Sci. Zool. **16**, 119-147.

[22] Ando N, Kanzaki R. 2016 Flexibility and control of thorax deformation during hawkmoth flight. Biol. Lett. **12**, 20150733. (10.1098/rsbl.2015.0733) 26740560 PMC4785917

[23] Lehmann FO, Dickinson MH. 1997 The changes in power requirements and muscle efficiency during elevated force production in the fruit fly *Drosophila melanogaster*. J. Exp. Biol. **200**, 1133-1143. (10.1242/jeb.200.7.1133) 9131808

[24] Combes SA, Gagliardi SF, Switzer CM, Dillon ME. 2020 Kinematic flexibility allows bumblebees to increase energetic efficiency when carrying heavy loads. Sci. Adv. **6**, eaay3115. (10.1126/sciadv.aay3115) 32076646 PMC7002132

[25] Bellini A, Yazidi A, Filippetti F, Rossi C, Capolino GA. 2008 High frequency resolution techniques for rotor fault detection of induction machines. IEEE Trans. Ind. Electron. **55**, 4200-4209. (10.1109/TIE.2008.2007004)

[26] van Roy J, de Baerdemaeker J, Saeys W, de Ketelaere B. 2014 Optical identification of bumblebee species: effect of morphology on wingbeat frequency. Comput. Electron. Agric. **109**, 94-100. (10.1016/j.compag.2014.09.014)

[27] Nasir N, Mat S. 2019 An automated visual tracking measurement for quantifying wing and body motion of free-flying houseflies. Measurement **143**, 267-275. (10.1016/j.measurement.2019.05.007)

[28] Rockstein M, Bhatnagar PL. 1966 Duration and frequency of wing beat in the aging house fly, *Musca domestica* L. Biol. Bull. **131**, 479-486. (10.2307/1539987) 5979662

[29] Roberts SP, Harrison JF, Dudley R. 2004 Allometry of kinematics and energetics in carpenter bees (*Xylocopa varipuncta*) hovering in variable-density gases. J. Exp. Biol. **207**, 993-1004. (10.1242/jeb.00850) 14766958

[30] Yu W, Zhou Y, Guo J, Wyckhuys KAG, Shen X, Li X, Ge S, Liu D, Wu K. 2020 Interspecific and seasonal variation in wingbeat frequency among migratory Lepidoptera in northern China. J. Econ. Entomol. **113**, 2134-2140. (10.1093/jee/toaa134) 32607536

[31] Willmott AP, Ellington CP. 1997 The mechanics of flight in the hawkmoth *Manduca sexta*. I. Kinematics of hovering and forward flight. J. Exp. Biol. **200**, 2705-2722. (10.1242/jeb.200.21.2705) 9418029

[32] Askew GN, Tregear RT, Ellington CP. 2010 The scaling of myofibrillar actomyosin ATPase activity in apid bee flight muscle in relation to hovering flight energetics. J. Exp. Biol. **213**, 1195-1206. (10.1242/jeb.034330) 20228356

[33] Josephson R, Ellington C. 1997 Power output from a flight muscle of the bumblebee *Bombus terrestris*. I. Some features of the dorso-ventral flight muscle. J. Exp. Biol. **200**, 1215-1226. (10.1242/jeb.200.8.1215) 9319067

[34] Gilmour KM, Ellington CP. 1993 In vivo muscle length changes in bumblebees and the in vitro effects on work and power. J. Exp. Biol. **183**, 101-113. (10.1242/jeb.183.1.101)

[35] Tu MS, Daniel TL. 2004 Cardiac-like behavior of an insect flight muscle. J. Exp. Biol. **207**, 2455-2464. (10.1242/jeb.01039) 15184517

[36] Tu MS, Daniel TL. 2004 Submaximal power output from the dorsolongitudinal flight muscles of the hawkmoth *Manduca sexta*. J. Exp. Biol. **207**, 4651-4662. (10.1242/jeb.01321) 15579560

[37] Stevenson RD, Josephson RK. 1990 Effects of operating frequency and temperature on mechanical power output from moth flight muscle. J. Exp. Biol. **149**, 61-78. (10.1242/jeb.149.1.61)

[38] Weis-Fogh T. 1973 Quick estimates of flight fitness in hovering animals, including novel mechanisms for lift production. J. Exp. Biol. **59**, 169-230. (10.1242/jeb.59.1.169)

[39] Ellington CP. 1984 The aerodynamics of hovering insect flight. III. Kinematics. Phil. Trans. R. Soc. Lond. B **305**, 41-78. (10.1098/rstb.1984.0051)

[40] Götz KG, Wandel U. 1984 Optomotor control of the force of flight in *Drosophila* and *Musca*: II. Covariance of lift and thrust in still air. Biol. Cybern. **51**, 135-139. (10.1007/BF00357927)

[41] Hollenbeck AC, Palazotto AN. 2013 Mechanical characterization of flight mechanism in the hawkmoth *Manduca sexta*. Exp. Mech. **53**, 1189-1199. (10.1007/s11340-013-9726-5)

[42] Casey C, Yager C, Jankauski M, Heveran CM. 2022 The flying insect thoracic cuticle is heterogenous in structure and in thickness-dependent modulus gradation. Acta Biomater. **138**, 422-429. (10.1016/j.actbio.2021.10.035) 34740857

[43] Gau J, Wold ES, Lynch J, Gravish N, Sponberg S. 2022 The hawkmoth wingbeat is not at resonance. Biol. Lett. **18**, 20220063. (10.1098/rsbl.2022.0063) 35611583 PMC9131119

[44] Dickinson MH, Lehmann FO, Chan WP. 1998 The control of mechanical power in insect flight. Am. Zool. **38**, 718-728.

[45] Casey C, Heveran C, Jankauski M. 2023 Experimental studies suggest differences in the distribution of thorax elasticity between insects with synchronous and asynchronous musculature. Figshare. (10.6084/m9.figshare.c.6472362) PMC10072941 37015268

